# Antimicrobial peptide Epinecidin-1 promotes complete skin regeneration of methicillin-resistant Staphylococcus aureus-infected burn wounds in a swine model

**DOI:** 10.18632/oncotarget.15042

**Published:** 2017-02-03

**Authors:** Han-Ning Huang, Chieh-Yu Pan, Hung-Yi Wu, Jyh-Yih Chen

**Affiliations:** ^1^ Marine Research Station, Institute of Cellular and Organismic Biology, Academia Sinica, Jiaushi, Ilan, Taiwan; ^2^ Department and Graduate Institute of Aquaculture, National Kaohsiung Marine University, Kaohsiung, Taiwan; ^3^ Department of Veterinary Medicine, College of Veterinary Medicine, National Pingtung University of Science and Technology, Neipu, Taiwan

**Keywords:** antimicrobial peptide, Epinecidin-1, methicillin-resistant Staphylococcus aureus

## Abstract

This report shows that the antimicrobial peptide (AMP) Epinecidin-1 (Epi-1) efficiently heals MRSA-infected heat burn injuries and provides protection from infection in a pig model. The presence of an optimal level of Epi-1 induces cell proliferation by promoting cell cycle progression through an increase in S-phase cells. Epi-1 also induces proliferation to cover the wounded region in an *in vitro* cell proliferation assay using immortalized human epithelial HaCaT cells. Next, the *in vivo* wound healing efficiency of Epi-1 was tested in heat-burned pig skin infected with MRSA under *in vivo* conditions. Treatment of the injury with Epi-1 for 1 h at six hours post-infection completely healed the wound within 25 days. Conversely, the injury in the untreated control was not healed 25 days post-infection. Histological staining of wound sections with H&E showed that Epi-1 enhanced vascularization and increased epithelial activities in the wound region. Neutrophil recruitment to the wounded region in the Epi-1-treated sections was visualized by Giemsa staining. Additionally, Masson's trichrome staining of wound sections confirmed that Epi-1 enhanced extracellular collagen compound formation. The induction of sepsis-associated blood C-reactive protein (CRP) and the pro-inflammatory cytokine IL-6 in response to MRSA infection was also suppressed in pigs that received Epi-1. Taken together, the results demonstrate that the biomaterial Epi-1 heals wounds through increasing epithelial cell proliferation, vascularization, and the formation of collagen and controls MRSA infection-mediated sepsis in pigs.

## INTRODUCTION

Infection of bodily injury sites causes morbidity in wounded patients [1]. Despite dramatic developments in trauma care and management, wound infections with pathogens such as methicillin-resistant *Staphylococcus aureus* (MRSA) are a major challenge to medical researchers [2]. The pathogen *Staphylococcus aureus* (SA) is commonly found on the skin and in the noses and throats of the long-term hospitalized and can cause infections through injuries [3]. Although most SA infections are minor, the infections sometimes lead to serious infections and mortality in people who are immunocompromised or have diabetes and MRSA exposure to blood [4]. The emergence of pathogens resistant to available antibiotics for skin wound infections is a major challenge in wound management, and the identification of suitable therapeutic alternative agents to existing antibiotics will minimize the emergence of drug resistance to single antibiotics in cases such as MRSA [5, 6]. Additionally, most systemic antibiotic agents possess poor tissue penetration capabilities in the wound region. Thus, topical administration at the wound region has the advantage of bypassing the need for an intact circulatory system [7, 8].

During drug screening, immortalized human adult keratinocyte cells (HaCaT) are widely used to investigate the *in vitro* proliferation and migration efficiency of epithelial cells to the wound region and the wound healing efficiency [9, 10]. Generally, extracellular calcium is an important factor in epithelial proliferation functions. The ‘calcium switch’ causes the cells to exit the cell cycle and commits them to terminal differentiation [11]. Hence, compounds that block the calcium switch-mediated differentiation of epithelial cells are desirable for epithelial proliferation [11]. Typically, suppression of the expression of the gap junction proteins Connexin (Cx) 43 and Keratin 3 (K3) favors cell proliferation and migration for wound healing [12–14].

Wound healing in humans involves a series of coordinated occurrences, including arrest of the hemorrhage, inflammatory responses, re-epithelialization, angiogenesis, recruitment of neutrophils, formation of granulation tissue and extracellular remodeling [15]. Thus, studying wound injury repair mechanisms or screening therapeutic candidates for healing in large animal models such as swine is physiologically more relevant to humans than a cell culture model [16, 17].

Epinecidin-1 (Epi-1) is an antimicrobial peptide (AMP) isolated from a marine organism. We previously isolated the gene encoding the AMP Epi-1 from a cDNA and genomic DNA library of a grouper (*Epinephelus coioides*). Structurally, Epi-1 is similar to pleurocidin, which is a protein from the winter flounder (*Pleuronectes americanus*) [18, 19] that exhibits reduced cytotoxic effects and antimicrobial functions against a range of key disease-causing pathogens, including MRSA under *in vitro* conditions and in rodent models [20–22].

This report demonstrates that Epi-1 promotes epithelial proliferation by progressing cell cycle pathways and promotes migration by regulating the expression of extracellular matrix complexes under *in vitro* conditions. The efficiency of Epi-1 in healing heat burn injury-mediated MRSA infections in swine is demonstrated in this report. Additionally, the induction of sepsis associated with MRSA infection is also reduced by Epi-1. Taken together, this report shows that Epi-1 is a potential candidate for therapeutic associated wound healing infection studies.

## RESULTS

### Epi-1 enhances keratinocyte cell proliferation and migration

**Figure 1:**
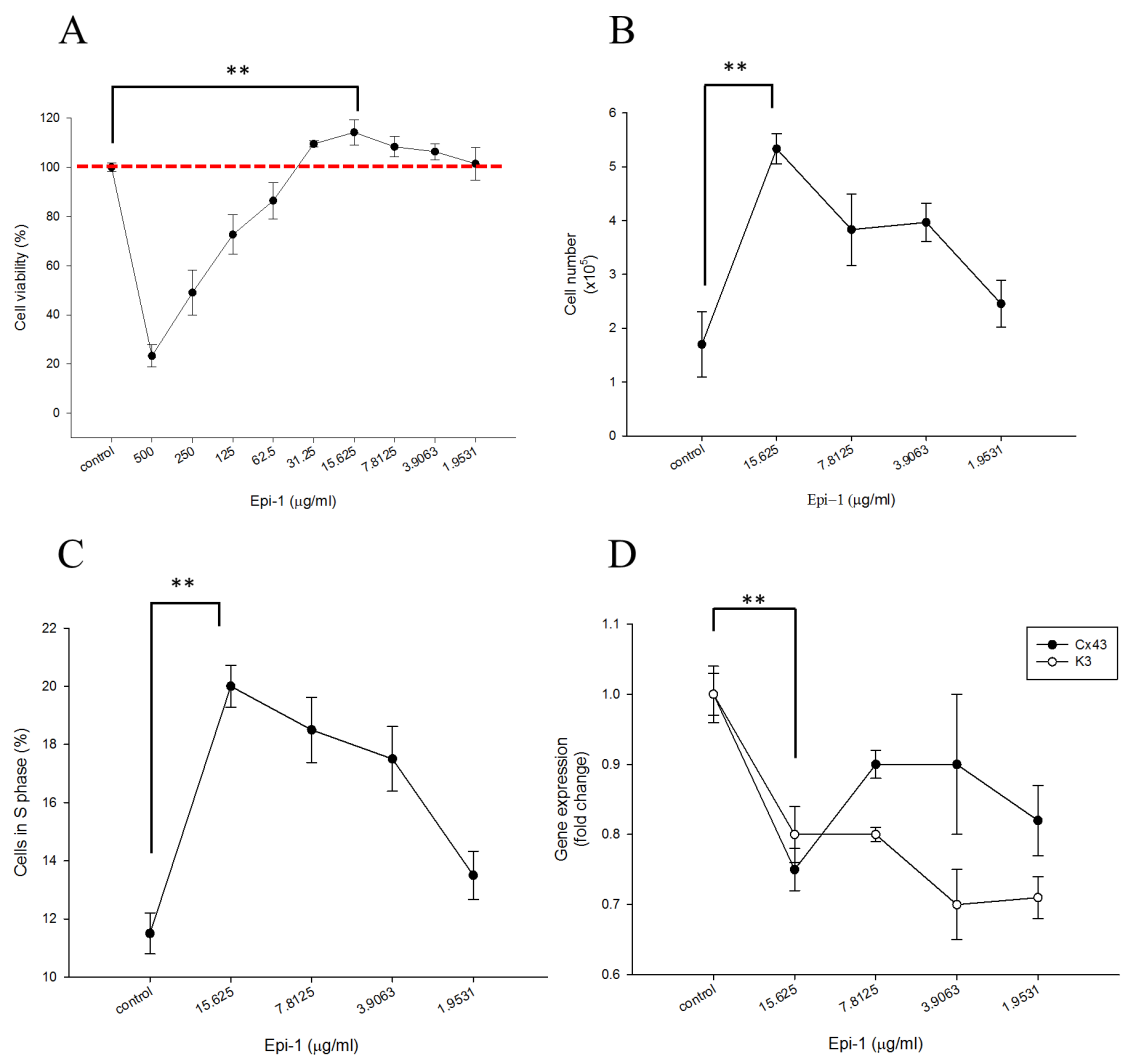
Epinecidin-1 (Epi-1) increases keratinocyte proliferation One day after culture, HaCaT cells were serum starved overnight. Subsequently, the cells were treated with 0 to 500 μg/ml Epi-1 for one hour, and then cultured with normal cell culture medium for 48 h. **A**. A cell viability assay was conducted with 20,000 cells/well seeded in a 96-well plate that had been treated with the PBS control (0 μg/ml) or Epi-1 at 500 μg/ml and two-fold serial dilutions to 1.9531 μg/ml for 1 h. The medium was replaced with normal medium and the cells were cultured for 48 h. **B**. Cells were seeded in 6-well plates at a density of 100,000 cells/well and treated with the PBS control (0 μg Epi-1) or 1.9531-15.625 μg/ml Epi-1 before counting. **C**. Cells that had been cultured in six-well plates and treated with or without Epi-1 were harvested, stained with propidium iodide, and subjected to flow cytometry to detect the percentage of S-phase cells. **D**. Total RNA was isolated from the cells that had been cultured in six-well plates and treated with or without Epi-1 and was used for the RT-PCR analysis to detect the expression of the differentiation marker K3 and Cx43. The expression was normalized to GAPDH, and the relative fold change is displayed.

**Figure 2:**
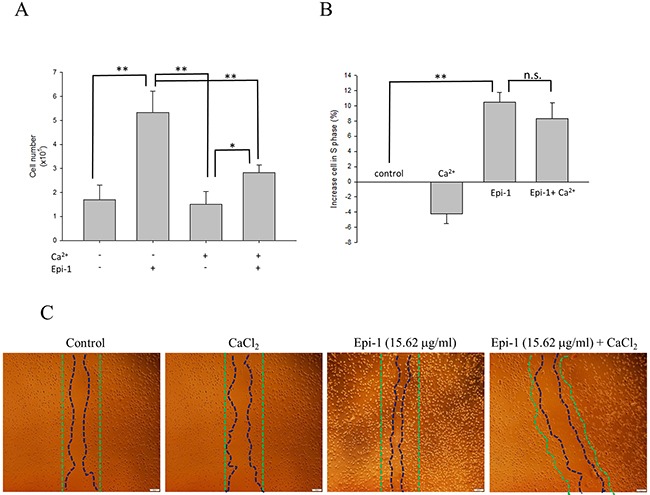
Epi-1 inhibits cell differentiation by extra cellular calcium and increases cell proliferation **A**. Approximately 100,000 HaCaT cells/well cultured in 6-well plates were serum-starved and treated with PBS alone (control), 15.62 μg/ml Epi-1 and/or 1.6 mM CaCl_2_, and the fraction of proliferating cells was assessed by counting the cells in a Countess automated cell counter. **B**. The percentage of cells from each group, i.e., PBS alone (control), 15.62 μg/ml Epi-1 and/or 1.6 mM CaCl_2_, in S-phase was obtained using a cytometer. **C**. Cells growing in a monolayer were scratched with a P1000 pipette tip to generate an artificial wound and then treated with 15.62 μg/ml Epi-1 in the presence or absence of 1.6 mM CaCl_2_. Cell migration and proliferation in the wounded region was calculated by assessing the photographed image using an image analyzer.

Epithelial cell proliferation and migration are important attributes of the wound healing process. Human immortalized keratinocyte HaCaT cells were treated with or without various concentrations of Epi-1, and cytotoxicity was assessed using a cell viability assay (Figure 1A). The presence of Epi-1 at a concentration up to 31.25 μg/ml did not affect the cell viability of the HaCaT cells, and the viability reached a maximum following treatment with 15.625 μg/ml of Epi-1 (Figure 1A). Hence, Epe-1 dosages up to 15.625 and its dilutions were used to elucidate the role of Epi-1 in keratinocyte cell proliferation and repair. HaCaT cells treated with 15.625 μg/ml of Epi-1 exhibited increased cell numbers and proportions of actively dividing S-phase cells out of the total cells (Figure 1B-1C). The expression of the epithelial negative cell surface markers connexin (Cx) 43 and keratin 3 (K3) was significantly decreased when the HaCaT cells were treated with 15.625 μg/ml of Epi-1 (Figure 1D). The migration of the HaCaT cells to cover the scratch created to mimic a wound in the *in vitro* wound healing assay was assessed to confirm the effect of Epi-1 on wound healing properties (Figure 2). Treatment with 15.625 μg/ml of Epi-1 promoted cell proliferation and migration into the wounded region, whereas extracellular calcium supplementation through calcium chloride (CaCl_2_), which is involved in terminal differentiation, prevented the wound healing property of Epi-1 (Figure 2A-2C).

### Epi-1 decreases MRSA counts at the wound injury site

**Figure 3:**
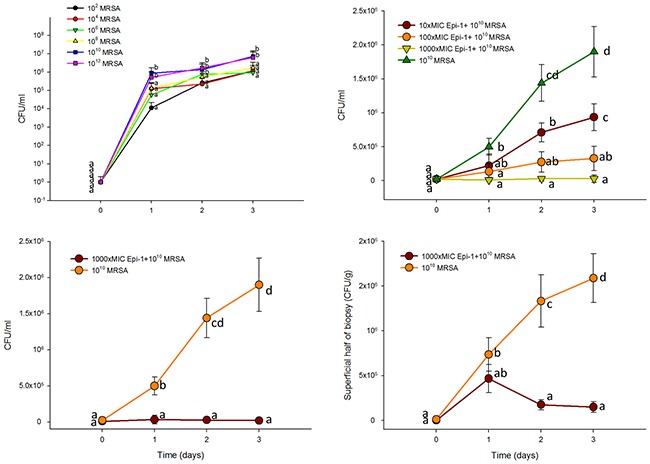
Epi-1 decreases the heat injury-mediated MRSA infection in pigs Three-centimeter-diameter heat injury wounds were generated using a heated aluminum bar after the pigs were fasted overnight and sedated. A MRSA suspension in 0.5 ml of saline was used to inoculate the wounded region. The wounded area was thoroughly washed before sampling, and then samples were collected. **A**. Wounds infected with MRSA alone at 10^2^ to 10^12^ CFU/0.5 ml. MRSA counts (CFU/ml) were calculated 1, 2 and 3 days post infection. **B**. At six hours post infection with 10^10^ CFU MRSA, the wounds were treated with Epi-1 concentrations equivalent to 0 (PBS only), 10 (90 μg/ml), 100 (900 μg/ml), 1000 (9 mg/ml)-fold of the MIC for MRSA for 1 h. Subsequently, the MRSA counts were estimated in surface wash sample concentrates. **C**. Bacteria counts in the surface wash concentrates. **D**. Concentrations of bacteria in the superficial and deep layers of the biopsies.

To assess the wound healing efficiency, a heat burn injury was created in a pig model, and the injured portion was treated with 10^2^ to 10^12^ CFU/ml of methicillin-resistant *Staphylococcus aureus* (MRSA). MRSA was enumerated in CFU/ml on 1, 2 and 3 days post-infection. The counts were highest (10^10^ and 10^12^ CFU/ml) in the infected wound samples at three days post-infection. Thus, the 10^10^ CFU/ml count was used for the analysis (Figure 3A). When the cells were treated with the 10 (90 μg/ml), 100 (900 μg/ml), and 1000 (9 mg/ml)-fold MIC equivalent doses of Epi-1 at six hours post-infection, the MRSA counts in the surface wash concentrated samples from the wound region were greatly decreased. Additionally, the samples treated with the 1000 (9 mg/ml)-fold MIC equivalent of Epi-1 showed no detectable MRSA counts (Figure 3B). Similarly, Epi-1 decreased the MRSA levels in the surface washes and superficial and deep halves of the biopsies (Figure 3C-3D).

### Epi-1 heals heat burn injuries infected with MRSA

**Figure 4:**
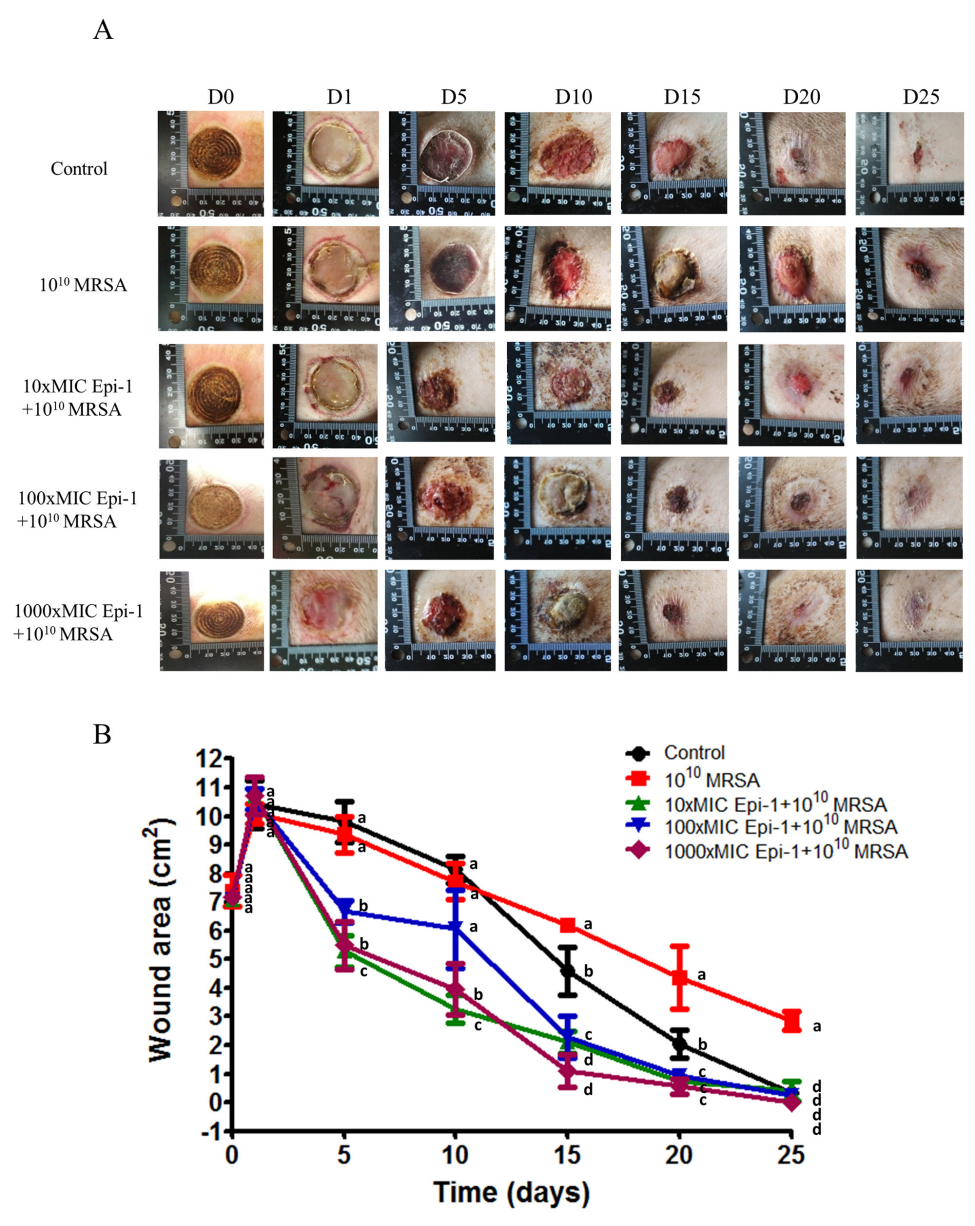
Epi-1 cures MRSA infection in heat burn wounds Three-centimeter-diameter heat wounds were created with a heated aluminum bar after the pigs fasted overnight and were sedated. A 10^10^ CFU MRSA suspension in 0.5 ml of saline was used to inoculate the wounded region. The wounded area was thoroughly washed before sampling, and then samples were collected. **A**. The MRSA infection-mediated wound healed after treatment with Epi-1 at 6 h post infection. **B**. The area of the wound injury was measured at 0, 1, 5, 10, 15, 20 and 25 days post infection using photo shot software (Scion, Frederick, MD), and the percentage contraction was calculated by dividing the initial wounding area on day 0 by the wounding area on different days.

Pigs with heat burn injuries infected with MRSA were maintained in the animal facility, and their normal activity, efficiency of wound healing, and severity of wound progression were recorded once daily for 25 days (Figure 3). In the MRSA-alone infected pigs, the wound was not healed even at 25 days post-treatment. In the pigs treated with Epi-1 at 6 h post-MRSA infection, the wound progression was significantly reduced and the wound was completely healed following treatment with the 1000 (9 mg/ml)-fold MIC equivalent Epi-1 dose (Figure 4). The wound area was completely healed in the pigs that received the 1000 (9 mg/ml)-fold MIC concentration of Epi-1 (Figure 4B).

### Epi-1 hastens epithelial layer formation and neutrophil recruitment to the wound region

**Figure 5:**
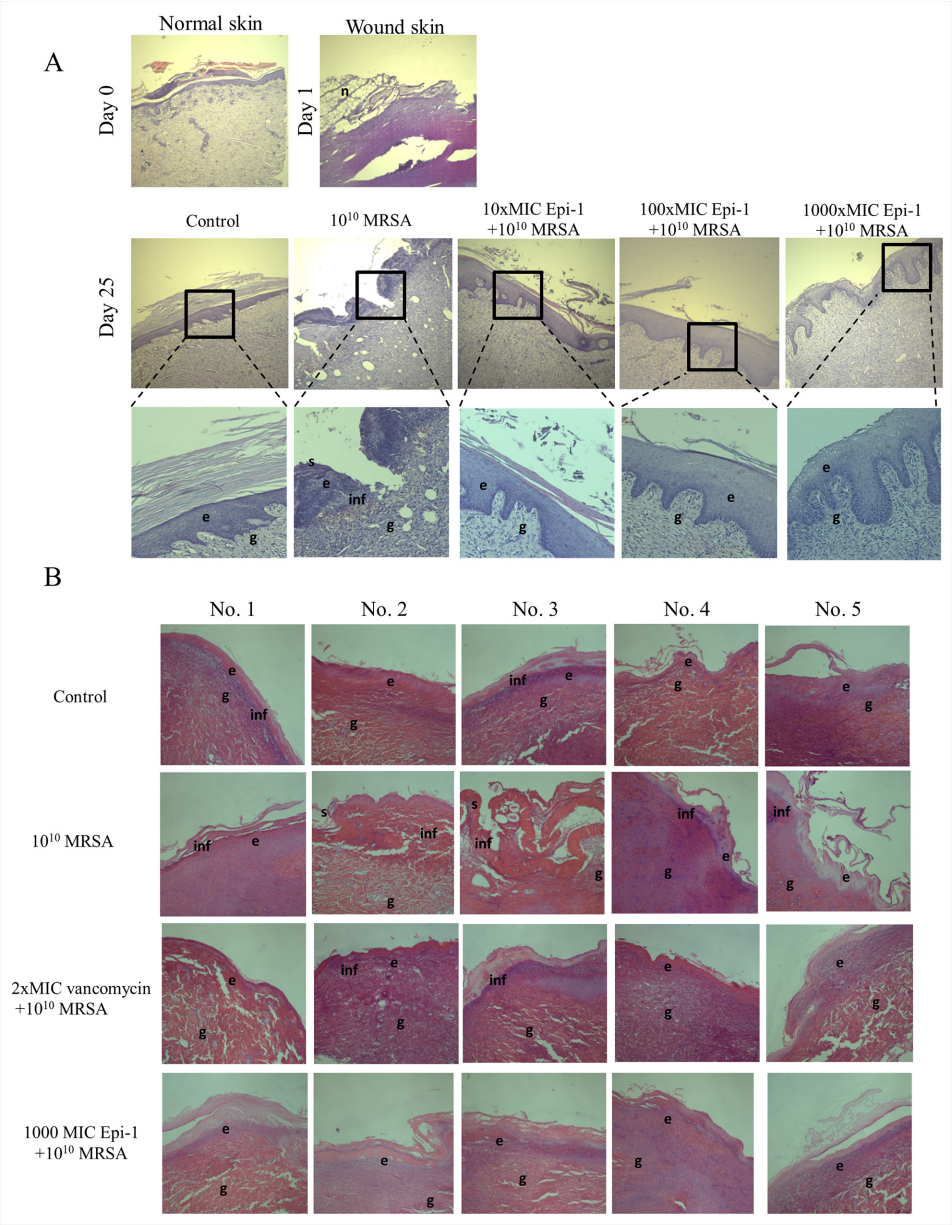
Epi-1 enhances wound healing activities in skin wounds Histological sections collected at the time of infection and various time points after infection were sectioned and stained with H&E, and the formation of the epithelial layer during the healing process was visualized. **A**. Enhanced vascularization was observed in the Epi-1-treated sections. **B**. Increased Notch activity was observed in epithelial cells from the Epi-1-treated samples.

**Figure 6:**
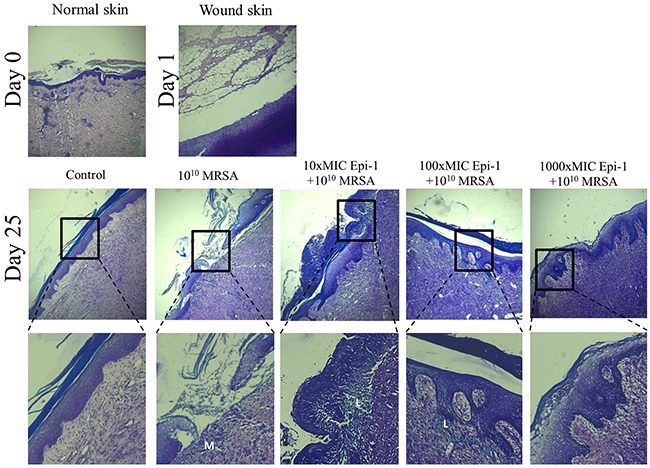
Epi-1 increases the recruitment of leukocytes near the injury region to accelerate wound healing Histological sections were collected at the time of infection and various time points after infection, sectioned, stained with Giemsa, and neutrophil recruitment was visualized. Sections from the Epi-1-treated samples showed an increase in the number of neutrophils near the infected region.

Histological sections taken from uninfected animals (normal skin) one day post-infection (day 1) and 25 days post-infection (day 25) were stained with H&E, and the epithelial activity was observed under a microscope (Figure 5). Histologically, an early inflammatory reaction was observed in the wound skin on day 1, wherein necrotic cells and inflammatory cells were observed. Additionally, epithelial regeneration appeared to be significantly different following treatment of the wounds with the various Epi-1 dosages. In the wounded skin, uniform epithelial coverage was clearly visualized after H&E staining, and the epithelial layer was disrupted in the day 1 and day 25 sections (Figure 5A). Wounds treated with the 1000-fold MIC concentration of Epi-1 recovered from the disruption of epithelial layer formation as a result of wound infection and capped the wound in the epithelial layer. Skin regeneration was also good in the vancomycin-treated group, although inflammatory cells were found in the organization (Figure 5B). Giemsa staining of the wound sections showed that the recruitment of neutrophils to the wounding site was enhanced in the pigs treated with Epi-1 (Figure 6). These macroscopic and microscopic observations under *in vivo* assessment suggested that the topical application of Epi-1 might have a favorable influence on the various phases of burn wound healing and thus accelerate the healing process.

### Epi-1 hastens the formation of extracellular matrix collagen around the wound region and decreases the sepsis associated with the induction of CRP-1 and IL-6

**Figure 7:**
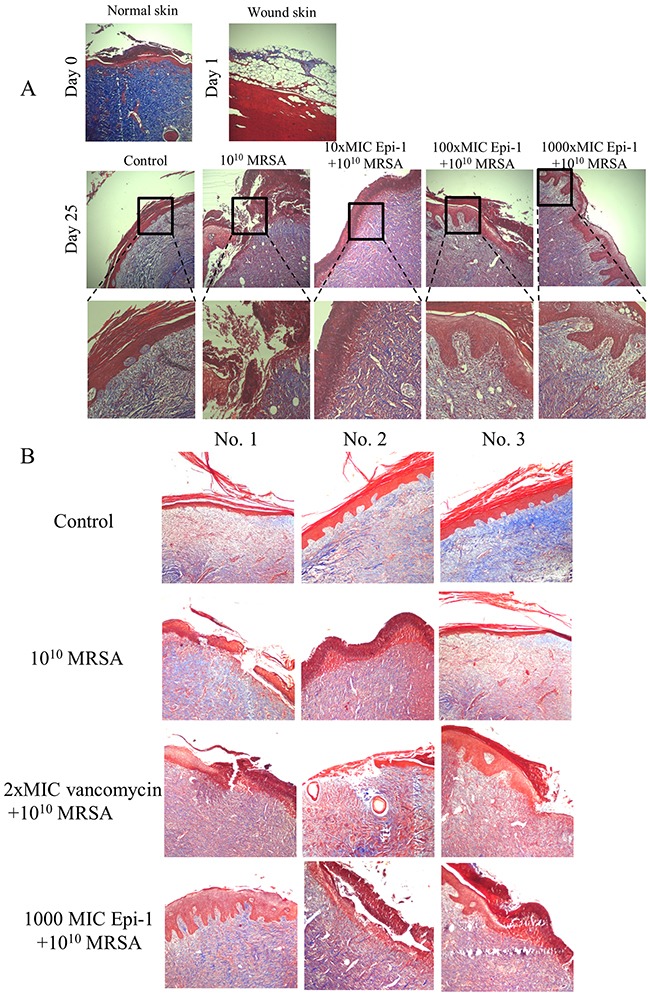
Epi-1 enhances the formation of collagen structures at the healing wound site Histological sections were collected at the time of infection and various time points after infection, sectioned, and subjected to Masson's trichrome staining to visualize collagen formation. **A**. The Epi-1-treated wounds showed an improvement in the deposition of the collagen layer. **B**. The deposition of the collagen layer in the Epi-1-treated samples was superior to the samples treated with the currently available MRSA antibiotic Vancomycin.

**Figure 8:**
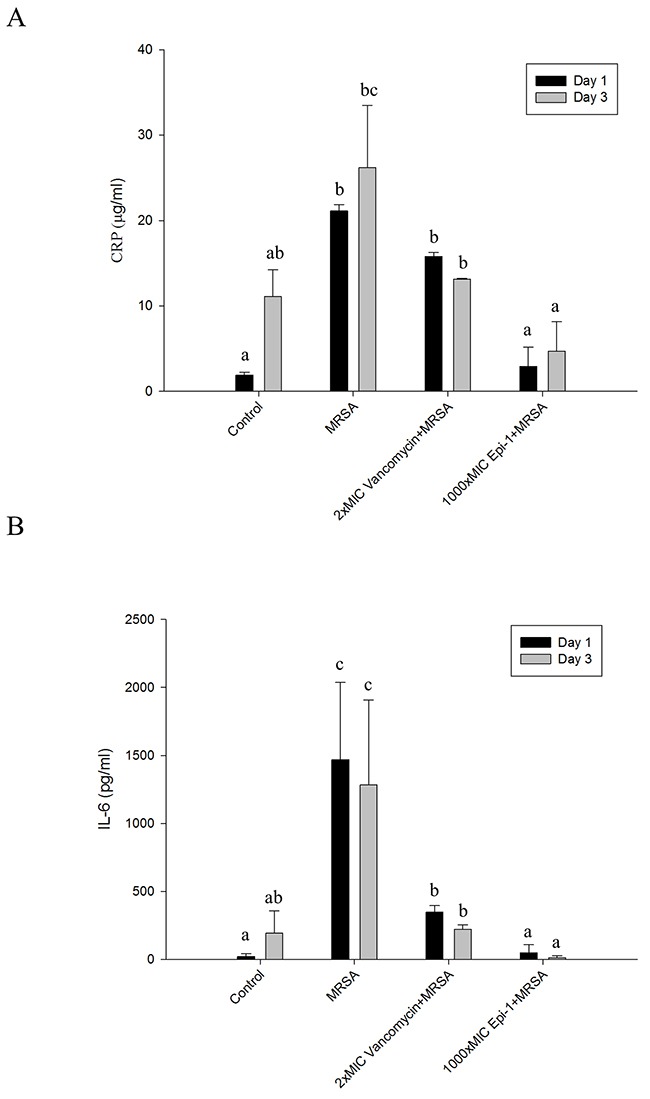
Epi-1 regulates the induction of C-reactive protein (CRP) and pro-inflammatory cytokine IL-6 expression associated with injury-mediated inflammation and sepsis Heat wound-injured pigs were infected with MRSA and treated with PBS (control), Vancomycin (0.5 mg/kg body weight) and a 1000-fold MIC of Epi-1 (9 mg/ml) at 6 h post infection. Blood was collected from the vein and immediately processed. **A**. Plasma was isolated from the blood and the CRP content was assayed with an ELISA. **B**. Serum IL-6 levels were assayed with an ELISA.

The sections were subjected to Masson's trichrome staining to visualize collagen formation (Figure 7). The formation of the collagen layer was completely disrupted in the MRSA-infected wounds, whereas Epi-1 treatment enhanced the formation of collagen around the wound region (Figure 7). Additionally, the efficiency of collagen formation in the Epi-1-treated wounds was superior to the presently available curative antibiotic vancomycin (Figure 7B). The levels of the MRSA infection-mediated inflammation-associated marker C-reactive protein (CRP) and the pro-inflammatory cytokine IL-6 were estimated in the plasma and serum using the ELISA technique (Figure 8). Pigs that received Epi-1 treatment in the wound region suppressed the CRP and IL-6 levels in the circulatory system, showing that the induction of inflammation by MRSA was neutralized by Epi-1 (Figure 8).

## DISCUSSION

MRSA infection constitutes a major therapeutic challenge for the management of burn wounds and chronic wounds [1]. Unlike normal wounds (i.e., surgical incision or superficial injury) that heal in an orderly manner, chronic wounds with an underlying pathological infection process prolong tissue injury and impair normal healing [23, 24]. The emergence of drug-resistant pathogens and the reduced tissue availability of administered systemic antibiotics contribute to the persistence of chronic wounds in one-fourth of the patients receiving antibiotic treatment [25]. Thus, the identification of novel candidates to heal wound injuries and infections by topical application is necessary to prevent chronic wounds. AMPs are short amino acid chain molecules that are involved in the modulation of the immune response and are efficient in eradicating a range of pathogens; thus, AMPs may be an alternative study material to identify novel drugs [26]. Epi-1, which is an AMP from a marine organism, has reported anti-microbial functions against MRSA and immunomodulatory functions with less cytotoxic effects in *in vitro* cell culture and mouse models [22].

This report demonstrated that Epi-1 promoted epithelial wound healing properties under *in vitro* conditions. When human immortalized keratinocyte HaCaT cells were treated with Epi-1, the AMP increased epithelial cell proliferation and migration (Figure 1). The presence of high extracellular calcium may signal epithelial cells to exit the cell cycle to reach terminal differentiation [11]. When Epi-1 was co-treated with calcium chloride, the calcium-mediated terminal differentiation was decreased by Epi-1 (Figure 2). Epi-1 modulation of the cell cycle may be associated with signaling pathways and increase the proportion of cells in the active “S” phase of the cell cycle (Figure 1C). Additional understanding of the modulation of cell cycle-associated genes by Epi-1 may help elucidate this mechanism. Epi-1 also suppresses the expression of connexin (Cx) 43 and Keratin 3 (K3), which influence the active migration of epithelial cells towards a wound region (Figure 1D).

Investigating wound healing mechanisms of novel drug candidates in large animal models with a well-established epithelial system is necessary to mimic wound healing in humans [27]. Pig models are suitable for investigations of wound injury and infection models, and their epithelial system is more relevant to humans [16, 17]. When heat burn wounds were infected with MRSA, the MRSA colony forming units were greatly increased in the wound (Figure 3A), whereas the topical application of Epi-1 at six hours post-infection greatly reduced the MRSA counts in the wound region (Figure 3). The heat burn wound treated with MRSA was not cured even after 25 days, but treatment with Epi-1 at the 1000 (9 mg/ml)-fold MIC concentration completely healed the wound (Figure 4). Thus, the direct antimicrobial function of Epi-1 may kill MRSA and prevent the injury from becoming a chronic wound.

A series of processes are essential during the wound healing process, including the formation of an epithelial layer, recruitment of neutrophils to the wound region, and the formation of extracellular matrix compounds, such as collagen [28]. In this report, the formation of an epithelial layer was demonstrated by visualizing epithelial cells in H&E-stained sections of the wound surface (Figure 5). Leukocyte recruitment to the injury site was demonstrated by Giemsa staining (Figure 6), and collagen formation was visualized by Masson's trichrome staining (Figure 7). In all cases, the topical application of the 1000 (9 mg/ml)-fold MIC equivalent of Epi-1 in the heat wound-infected region showed efficiency in hastening the wound healing process. Additionally, the induction of sepsis was observed based on the elevated levels of the sepsis-associated marker C-reactive protein (CRP) and the pro-inflammatory cytokine IL-6 in the circulatory system in pigs with MRSA-infected wounds (Figure 8). In contrast, the CRP and IL-6 levels were under control in the pigs treated with Epi-1 after infection (Figure 8).

Taken together, our results demonstrated that the Epi-1 peptide promotes epithelial wound healing under *in vitro* conditions and effectively heals MRSA-infected wounds in a pig model.

## CONCLUSIONS

1. We have demonstrated that Epi-1 exerts potent antimicrobial activity against methicillin-resistant *Staphylococcus aureus* (a MDR clinical isolate). The use of Epi-1 may complement the use of antibiotics. AMPs are unlikely to induce resistance.
2. Treatment with Epi-1 significantly reduces MRSA infection in a mouse model of wound infection compared to untreated controls and mice treated with conventional antibiotics. Given the prophylactic efficacy of Epi-1 and its inability to engender resistance, this AMP may be suitable for situations in which there is a high risk of infection.
3. Our model is valuable for future research on the pathophysiology of wound healing and testing new therapeutics for the treatment of bacterial infections during wound healing.

## MATERIALS AND METHODS

### Animals, antimicrobial peptide and the methicillin-resistant *Staphylococcus aureus* strain

Six-week-old clinically healthy Yorkshire breed pigs with an average body weight (BW) of 10 to 13 kg were purchased from a commercial pig herd (Pingtung, Taiwan). Prior to the experiment, the pigs were acclimatized in animal housing facilities and subjected to 12 h of starvation. The absence of clinical signs of disease was confirmed as reported previously [29]. The pigs were anesthetized by intramuscular (*i.m*.) injection with a mixture of 2 mg/kg body weight of xylazine (Balanzine, Health-Tech Pharmaceutical Co., Taipei, Taiwan) and 20 mg/kg body weight of ketamine (Imalgene 1000, Merial Taiwan Co., Taipei, Taiwan). All animal handing procedures were in accordance with the National Pingtung University of Science and Technology (NPUST), and all procedures were approved by the Animal Care and Use Committee of NPUST. All surgical procedures, including the insertion of catheters, injections, withdrawal of blood, and aliquoting of blood, were performed under aseptic conditions using 70% ethanol as a disinfectant. Amidated C-terminal Epinecidin-1_22–42_ (Epi-1) possessing the amino acid sequence GFIFHIIKGLFHAGKMIHGLV-NH2 was synthesized by GL Biochemistry (Shanghai, China). The synthetic peptide was reconstituted in phosphate-buffered saline (PBS; pH 7.4) to the desired concentrations for the experiments [22]. The MRSA strain is a clinical isolate from stool obtained from Taipei City Hospital (Heping Fuyou Branch). This strain is resistant to ampicillin, methicillin, oxacillin, and ciprofloxacin. The culture and quantification were performed as previously described [21, 22].

### Keratinocyte cell proliferation assay

The human immortalized keratinocyte cell line HaCaT was maintained in Dulbecco's modified Eagle's medium (DMEM) supplemented with 10% fetal bovine serum and 1% antibiotics (10,000 units/ml of streptomycin and 10,000 units/ml of penicillin) at 37°C with 5% CO_2_. Approximately 2×10^4^ cells/well were grown in 96-well plates and serum starved overnight. Then, the cells were treated with different Epi-1 dosages for 1 h, followed by incubation with normal medium for 48 h to test cell viability using the CellTiter 96 Aqueous One Solution assay (Cat. No. G3580, Promega, WI, USA). In the cell proliferation assay, HaCaT cells at a density of 1×10^5^ cells/well were cultured overnight in a six-well plate, serum starved for 24 h, treated with different concentrations of Epi-1 for 1 h and then incubated with normal medium for 48 h. Subsequently, the cells were harvested and counted in a cell counter or using the Tali^®^ cell cycle kit (Cat. No. A10798, Thermo Fisher Scientific, CA, USA) for Tali^®^ Image-Based cytometer (Cat. No. T10796, Thermo Fisher Scientific, CA, USA) analysis to evaluate the cell cycle.

### RT-PCR assay

Gene-specific primers for the keratinocyte markers Cx43 (F’- CCTTCTTGCTGATCCAGTGGTAC and R’- ACCAAGGACACCACCAGCAT; 154 bp) and K3 (F’-GGCAGAGATCGAGGGTGTC and R’-GTCATCCTTCGCCTGCTGTAG; 145 bp) were used to evaluate gene expression by RT-PCR. HaCaT cells at a density of 1×10^5^ cells/well were cultured overnight in a 6-well plate, serum starved for 24 h, treated with Epi-1 for 1 h and incubated with normal medium for 48 h. Then, the cells were harvested, and total RNA was extracted using an RNeasy mini kit (Cat. No. 74106, QIAGEN, Manchester, UK). Reverse transcription into cDNA was performed with the iScript cDNA Synthesis Kit (Cat. No. 1708891, BIO-RAD, Hercules, CA, USA) following the manufacturer's recommendations. The Fast-Run 2x Taq Master Mix (Cat. No. PT-TMM-AD, Bio-Protech, Taipei, Taiwan) was used to prepare the PCR master mix containing cDNA and the primer components in accordance with minor modifications to the vendor protocol. To detect keratinocyte differentiation, we assessed the Cx43 and K3 gene expression levels. DNA amplification was performed in a Veriti thermal cycler (Cat. no. 4375305, Thermo Scientific, CA, USA) with the following thermal cycling profile: an initial denaturation at 94°C for 5 min, followed by 30 cycles of amplification (denaturation at 94°C for 30 sec, annealing at 55°C for 30 sec, and extension at 72°C for 1 min) and a final extension at 72°C for 5 min. Expression was normalized to GAPDH, and the relative fold change was reported.

### HaCaT cell differentiation assay

We investigated whether Epi-1 could inhibit the HaCaT differentiation triggered by elevated extracellular calcium, which is a potent terminal differentiation signal that corresponds with the presence of increased Ca^2+^ in keratinocytes. The capacity of HaCaT cells to migrate, proliferate, and close a defined injury area into a confluent monolayer was evaluated by following a previously reported assay with some modifications [30]. Briefly, monolayer cells were scratched vertically with a P1000 pipette tip to create an artificial wound and washed twice with PBS to remove cellular debris. Then, the cells were treated with 15.62 μg/ml of Epi-1 in the presence or absence of 1.6 mM CaCl_2_. An equivalent volume of PBS was added in the untreated control wells. The scratched areas were photographed every 24 h, and the area containing migrating keratinocytes was calculated with Image-Pro Plus 6.0. The percent migration at the indicated time point was calculated by subtracting the total area of the scratch at the corresponding time point from the total area of the scratch at 0 h divided by the total area at 0 h multiplied by 100.

### Hot plate wound induction and MRSA infection

Each group included five pigs. In anesthetized pigs, the hair over the dorsum was clipped using an Osler blade, and the skin was subsequently scrubbed with a non-antibiotic-containing soap. Prior to wounding, normal skin specimens were collected from each location with a 4 mm-diameter biopsy punch. Six 3-cm-diameter wounds were created on the back in each animal with at least 3-cm intervals between wounds. The wounds were created in four columns parallel to the dorsal midline, with two columns on each side of the dorsal thoracolumbar midline. Contact burns were applied paravertebrally under aseptic conditions by placing a heated aluminum bar onto the dorsum of the animal. The aluminum bar was heated to 200°C with a Meeker gas burner. The core temperature of the bar was monitored with a digital thermometer. The heated bar was placed on the animal for 30 s. Application pressure was measured with a 50-ml syringe attached to the aluminum bar via a heat insulation unit. This device was designed to exert a pressure of 0.4 kg/cm_2_ when the piston was pushed into the barrel of the syringe with one hand from the “20 ml” to the “10 ml” mark while the other hand was holding the heat insulation unit to prevent any additional pressure on the heat transfer bar. Burn sites of approximately 10-12 cm_2_ each were made on the dorsum of the animal with 3 cm between each site or from the spine. The total burn size did not exceed 15% of the entire body surface area. Then, the wound was debrided the next day. Each wound was inoculated with 500 μl of sterile 0.9% saline with 0.05% Triton X-100 containing 10-10^12^ CFU of MRSA. At 6 h after inoculation, Epi-1 at the 10 (90 μg/ml), 100 (900 μg/ml), and 1000 (9 mg/ml)-fold MICs and vancomycin at the 2 (9 μg/ml)-fold MIC (dissolved in phosphate-buffered saline [PBS]) were applied in a total volume of 0.5 ml. One hour post-treatment, the wounds were covered with Tegaderm (3M, St. Paul, MN, USA) to maintain uniformity and prevent the pig from removing the treatments. The wounds were dressed every 3 days, and the different Epi-1 dosages were added until the 25th day. Samples were subsequently collected on 0, 1, 2, 3, 5, 10, 15, 20 and 25 days for histopathology analysis and microbiological bacterial counting.

### Quantification of the wound area

Wound healing was monitored up to 25 days and macroscopically recorded by photography. The contraction was recorded by measuring the wound area during healing. Correction for the growth of the animals was accomplished by determining the change in a larger area surrounding the wound, which was marked by a tattooed grid. Biopsies were taken at different time intervals to study wound healing parameters, such as epithelialization, extracellular matrix formation, collagen remodeling, and cell density.

### Wound sampling for MRSA counts

The wound surface was thoroughly washed with a swab soaked in 0.9% saline. Two sampling methods were used. In the first method, a rigid cylinder was pressed watertight against the wound surface. One milliliter of phosphate buffer with 0.1% Triton X-100 was pipetted into the cylinder, the wound surface was rubbed for 1 minute, and the cylinder contents were aspirated. This procedure was performed on two random sites in the wound. The final aspirate volume was determined, and the samples were added to empty Petri dishes and mixed with melted mannitol salt agar (MSA) (Cat. No 7143, NEOGEN, MI, USA) supplemented with oxacillin (Cat. No. 28221, Sigma Chemical Co, MO, USA) at a 2 μg/ml concentration. The Petri dishes were incubated at 37°C for 48 h, and the viable bacterial counts/ml were enumerated. In the second method, a cylindrical punch with a stopping piston from the shearing edge was rotated into the wound tissue. After removal, the tissue cylinder was excised at its deep base. The biopsies were taken at three sites in the wound. The sample sites were spaced approximately equidistant over the wound surface, and sites with lesions from previous biopsies were avoided. The biopsies were weighed, homogenized, and diluted. The quantity of bacteria was determined as the CFU per 0.5 ml of inoculum, per gram of biopsy, or per milliliter of surface wash fluid.

### Quantification of the plasma IL-6 and serum C-reactive protein levels

Plasma was generated by centrifugation of heparin-stabilized blood samples in an endotoxin-free vial. Centrifugation was performed immediately after blood collection. The plasma samples were kept at 4°C for a maximum of 1 h and then stored at -80°C. The IL-6 content was determined in the plasma using an abcam ELISA (Cat. no. ab100755, abcam, MA, USA). The C-reactive protein (CRP) content in the serum was determined using an Abnova ELISA (Cat. no. KA1920, Abnova, CA, USA) following the vendor's instructions.

### Histochemical analysis

Tissue samples from the wound edges were fixed for 24 h in 4% formaldehyde in PBS. After fixation, the tissue specimens were processed through graded concentrations of ethanol and xylene, embedded in paraffin wax, and cut at an 8-10-μm thickness. Then, the sections were rehydrated and stained with H&E, Giemsa, and Masson Trichrome staining for histological analysis.

### Statistical analysis

Univariate analysis of variance (ANOVA) performed with the SPSS statistical software 18.0 (SPSS Inc., Chicago, IL, USA) was used to identify significant differences between treatments. The error bars represent the standard error of the mean (SEM). Differences were defined as significant at a *P* value <0.05 or < 0.01. Different letters indicate significant differences between groups, whereas the same letter indicates no difference between groups. Statistical comparisons of the wound area data with the MRSA group were performed using Student's *t*-test at each time point.

## Abbreviations

Epi-1: Epinecidin 1
MRSA: Methicillin-resistant *Staphylococcus aureus*
SA: *Staphylococcus aureus*
CRP: C-reactive protein
